# Detection of a Water-Soluble Hypericin Formulation in Glioblastoma Tissue with Fluorescence Lifetime and Intensity Using a Dual-Tap CMOS Camera System

**DOI:** 10.3390/diagnostics14212423

**Published:** 2024-10-30

**Authors:** Mario Mischkulnig, David Reichert, Lionel Wightman, Vanessa Roth, Marijke Hölz, Lisa I. Körner, Barbara Kiesel, Djenana Vejzovic, Gabriel A. Giardina, Mikael T. Erkkilae, Angelika Unterhuber, Marco Andreana, Beate Rinner, Andreas Kubin, Rainer Leitgeb, Georg Widhalm

**Affiliations:** 1 Department of Neurosurgery, Medical University Vienna, 1090 Vienna, Austria; 2 Department of Medical Physics, Medical University of Vienna, 1090 Vienna, Austria; 3 Christian Doppler Laboratory OPTRAMED, Medical University Vienna, 1090 Vienna, Austria; 4 Hypericum LifeScience GmbH, 1230 Vienna, Austria; 5 Division of Biomedical Research, Medical University of Graz, 8010 Graz, Austria; 6 BioTechMed Graz, 8010 Graz, Austria

**Keywords:** hypericin, HHL-PVP, glioblastoma, fluorescence, visualization, fluorescence lifetime

## Abstract

Background: High hypericin-loaded polyvinylpyrrolidone (HHL-PVP) constitutes a novel approach to utilize the promising characteristics of hypericin for photodynamic diagnosis (PDD) and therapy (PDT) of brain tumors in an orally bioavailable formulation. The aim of this study was to investigate the ability of a Complementary Metal-Oxide-Semiconductor (CMOS) camera-based fluorescence imaging system to selectively visualize HHL-PVP in glioblastoma tissue even in the presence of 5-Aminolvevulinic acid (5-ALA) induced fluorescence, which is widely utilized in brain tumor surgery. Methods: We applied a previously established system with a non-hypericin specific filter for 5-ALA fluorescence visualization and a newly introduced hypericin-specific filter at 575–615 nm that transmits the spectrum of hypericin, but not 5-ALA fluorescence. Glioblastoma specimens obtained from 12 patients (11 with preoperative 5-ALA intake) were ex vivo incubated with HHL-PVP. Subsequently, fluorescence intensity and lifetime changes using both the non-hypericin specific filter and hypericin-specific filter were measured before and after HHL-PVP incubation and after subsequent rinsing. Results: While no significant differences in fluorescence signal were observed using the non-hypericin specific filter, statistically significant increases in fluorescence intensity (*p* = 0.001) and lifetime (*p* = 0.028) after HHL-PVP incubation were demonstrated using the hypericin-specific filter. In consequence, specimens treated with HHL-PVP could be identified according to the fluorescence signal with high diagnostic sensitivity (87.5%) and specificity (100%). Conclusions: Our CMOS camera-based system with a hypericin-specific filter is capable of selectively visualizing hypericin fluorescence in glioblastoma tissue after ex vivo HHL-PVP incubation. In the future, this technique could facilitate clinical investigations of HHL-PVP for PDD and PDT while maintaining the current standard of care with 5-ALA guidance.

## 1. Introduction

Primary central nervous system tumors are associated with significant morbidity and mortality, and show an annual incidence rate of 24.83 per 100,000 person-years [1]. While maximum safe resection is the treatment of choice in most of these tumors, this surgical goal is frequently unachievable due not only to the proximity to relevant functional brain areas but also to insufficient intraoperative visualization of the tumor and its margin [2]. Thus, a number of different tumor visualization tools for optimized intraoperative guidance have been established, including neuronavigation, ultrasound, intraoperative magnetic resonance imaging (MRI), and fluorescence guided resection [3,4]. Especially for the most aggressive primary brain tumor subtype, the glioblastoma, fluorescence-guided resection using 5-aminolevulinic acid (5-ALA) is nowadays a well-established application of photodynamic diagnosis (PDD) in neurosurgery [5,6]. Besides powerful tumor visualization, a major benefit of this fluorescent dye is that it is orally bioavailable [7]. After oral intake, the pro-drug 5-ALA is metabolized to protoporphyrin IX (PpIX), which can be visualized under blue light due to its characteristic fluorescence at a wavelength peak of approximately 635 mm [8,9]. In addition to conventional intensity-based fluorescence imaging, advanced systems that analyze fluorescence lifetime rather than intensity have shown promise in enhancing visualization, particularly for weak fluorescence signals, resulting in improved sensitivity [10].

While 5-ALA demonstrated the capacity to reliably visualize specific brain tumor entities such as glioblastoma and meningioma, other tumors, such as most low-grade glioma, cannot currently be reliably detected by 5-ALA fluorescence [11,12,13,14]. Given the poor prognosis of malignant brain tumors even with optimal treatment, photodynamic therapy (PDT) constitutes an important field of research to utilize fluorescent agents not only for diagnostic but also for therapeutic use [15,16]. While there are promising preliminary results suggesting that PDT techniques relying on 5-ALA are safe and may improve progression-free survival, their particular effect on tumor cells at some distance from the surface is probably limited due to the comparatively superficial tissue penetration depth of the required short wavelength excitation light of approximately 1 mm [17,18].

Aside from 5-ALA, which currently represents the most powerful and widely used fluorescence dye in routine neurosurgical practice, other fluorescent dyes, such as fluorescein and indocyanine green, have also been investigated in recent years [19,20]. A major drawback of most of these agents is that they primarily visualize blood–brain barrier deficiencies rather than tumor metabolism [21]. A particularly promising fluorescent agent is hypericin, a naturally occurring agent extracted from St. John’s Wort that has been shown to actively accumulate in brain tumor tissue [22,23,24]. An initial study of this new fluorescence agent demonstrated overall safety as well as favorable diagnostic accuracy for glioblastoma tissue, with a high sensitivity of 91–94% and a specificity of 90–100% [22]. While no clinical investigations of hypericin as a fluorescent dye have meanwhile been published, fluorescence lifetime was shown to significantly increase in glioblastoma tumor spheroids treated with the agent [24]. Furthermore, sufficient hypericin fluorescence excitation has been demonstrated across a broad wavelength spectrum ranging from ultraviolet light up to 590 nm, thus including spectra with comparatively deep tissue penetration and potentially suggesting that the agent may be a promising candidate not only for tumor visualization but also for PDT [25,26].

A drawback of previous hypericin formulations is their poor oral bioavailability, which necessitates intravenous administration [22]. To address this limitation, water-soluble formulations of hypericin attached to polyvinylpyrrolidone (PVP) were investigated to facilitate the water solubility of the fluorescent compound [26]. Most recently, marked improvements to this technique resulted in the implementation of high hypericin-loaded PVP (HHL-PVP), which allows a substantially increased hypericin content of approximately 40% as compared to only 1–2% in earlier formulations [27]. In principle, the visualization of the fluorescence maximum of HHL-PVP at 595 nm should be possible with conventional filter systems used for the detection of 5-ALA-induced PpIX fluorescence (wavelength peak at 635 nm) with long-pass filters at 440 nm [28,29]. In order to use the benefits of different fluorescence dyes, a reliable distinction between both fluorescence signals, including 5-ALA and HHL-PVP, is of major importance to optimize intraoperative tumor visualization. By the development of reliable and readily available techniques for selective HHL-PVP visualization, we expect that tumor visualization might also be feasible in cases with the presence of a visible 5-ALA fluorescence signal.

The aim of the present study was thus to investigate the capability of a previously developed dual-tap Complementary Metal-Oxide-Semiconductor (CMOS) camera-based system for real-time wide-field fluorescence lifetime imaging [10] to selectively visualize HHL-PVP in glioblastoma tissue specimens after ex vivo incubation in HHL-PVP solution.

## 2. Materials and Methods

In the present study, we performed fluorescence analyses in prospectively collected tissue specimens obtained during surgery before incubation and after incubation, as well as after incubation and subsequent rinsing of the specimens in saline solution. Tissue collection was performed in patients undergoing resection for suspected glioblastoma at the Department of Neurosurgery at the Medical University of Vienna between August 2023 and June 2024. Only patients with postoperative confirmation of a glioblastoma, specifically central nervous system (CNS) World Health Organisation (WHO) grade 4 and isocitrate dehydrogenase (IDH) wildtype, were included in the final analysis. The study was approved by the ethics committee at the Medical University of Vienna (EK418/2009—amendment), and patients provided informed consent.

### 2.1. Fluorescence Quantification Device

Imaging was performed with a dual-tap CMOS camera (pco.flim, PCO AG, Kelheim, Germany) as described in previous publications [10,30,31]. The only change made to the imaging setup was the exchange of the laser source to a 405 nm MatchBox Laser (150 mW, Integrated Optics, Vilnius, Lithuania), which required the development of a custom circuit board to control the laser with the camera system [10]. While the previously employed filter setup was optimized for the protoporphyrin IX fluorescence typically observed in 5-ALA-guided surgery using a bandpass filter with a relatively wide wavelength spectrum (665/150 BrightLine HC Semrock, DEX Health & Science LLC, Rohnert Park, CA, USA, “non-hypericin-specific filter”), it was assumed that this setup should be capable of visualizing hypericin fluorescence at its typical fluorescence emission maximum of 592 nm wavelength [26]. Initial analyses with this filter verified obvious increases in the fluorescence signal after HHL-PVP incubation in cases without 5-ALA administration.

**Figure 1:**
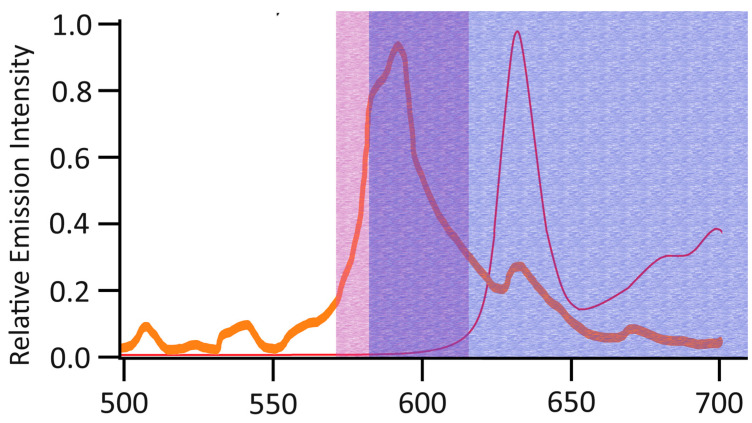
Schematic overview of relative emission intensity of hypericin (orange line) and PpIX fluorescence (red line) as well as the transmission spectra of the bandpass filters used in this study. The wavelength spectrum transmitted by the hypericin-specific filter is characterized by the red area and the wavelength spectrum transmitted by the hypericin-specific filter by the blue area while the purple area corresponds to the overlap of wavelengths that is transmitted by both filters. Adapted from fluorescence spectra for hypericin and PpX published by Kubin et al., 2008 and Minawikawa et al., 2016, respectively [26,32].

In addition, we aimed to optimize the system in order to allow for selective detection of hypericin fluorescence even in the presence of 5-ALA-induced PpIX fluorescence. We therefore upgraded the system with a narrower bandpass filter (595/40 ET, Chroma Technology Corp., Bellows Falls, VT, USA; “hypericin-specific filter”) that was tailored to the hypericin fluorescence peak at 595 nm, but which blocked the fluorescence caused by 5-ALA-induced PpIX accumulation at 620–720 nm [32]. A schematic overview of the relative emission intensity of hypericin and PpIX fluorescence as well as the transmission spectra of both bandpass filters is provided in Figure 1. It is of note that the low autofluorescence signals before incubation in the HHL-PVP solution observed in initial experiments led to increased background noise in these measurements. We accounted for this using a threshold-based denoising approach for the fluorescence lifetime analysis.

### 2.2. Specimen Collection, Processing and Analysis

During resection of suspected glioblastoma, we generally administered 5-ALA (20 mg kg^−1^ body weight) approximately 3 h before anesthesia for intraoperative guidance. In this study, tissue specimens were collected, and the visible 5-ALA fluorescence status (strong, vague, or no fluorescence) was documented for each specimen by the performing neurosurgeon. Specimens were then immediately divided into one part that was used for the fluorescence experiments presented in this study, while the other part was sent for histopathological analysis according to the current fifth WHO Classification of Tumors of the Central Nervous System published in 2021 [33]. The specimens for fluorescence analyses then were immediately analyzed (status before incubation) and subsequently incubated in a 4-molar HHL-PVP solution. After incubation for 1 h at room temperature, the specimens were subjected to a second fluorescence analysis (status after incubation). Specimens were then rinsed in physiological saline for 10 min in order to verify that the obtained fluorescence signal corresponded to actual HHL-PVP accumulation within tumor tissue rather than residual HHL-PVP solution on the specimens, and then a final fluorescence analysis (status after rinsing) was carried out.

### 2.3. Statistical Analysis

The statistical analyses and creation of figures were performed using SPSS statistical software (Version 28.0, IBM Inc., Armonk, New York, NY, USA). In the first step, differences between the time points (before incubation, after incubation, and after rinsing) were analyzed using Analysis of Variance (ANOVA). In the case of an overall difference between time points being detected, a second step designed to analyze the differences between distinct time points using paired *t*-tests was employed. In a final third step, if significant differences in fluorescence intensity and/or lifetime were detected in the previous univariate analyses for one of the filter systems, a binary regression model was performed in order to predict measurements that had been exposed to HHL-PVP (time points after incubation and after rinsing) as compared to specimens not treated with HHL-PVP. Subsequently, a receiver operating characteristic (ROC) analysis with this regression model was performed to estimate the achievable discrimination between specimens exposed to HHL-PVP and those before incubation. The threshold of statistical significance was set at the commonly applied value of *p* = 0.05.

## 3. Results

In the present study, we included 12 patients who underwent surgery for a histopathologically confirmed glioblastoma, CNS WHO grade 4, IDH wildtype. The median patient age at time of surgery was 62 years old (range: 48–77 years), with male and female patients accounting for 6 cases (50%) each.

### 3.1. 5-ALA Fluorescence Administration

In 11 patients, a standard dose of 5-ALA was administered prior to surgery. In all patients with preoperative 5-ALA administration, strong fluorescence was detected during surgery as the maximum fluorescence level. The remaining patient did not receive 5-ALA prior to surgery due to urgent surgery and transfer before surgery from another department. In the specimens collected after preoperative 5-ALA administration, 9 specimens (81.8%) showed strong fluorescence and two specimens showed vague fluorescence (18.2%) during surgery according to the neurosurgeons’ intraoperative assessment. The 11 specimens with visible 5-ALA-induced fluorescence and the one sample from a patient without preoperative 5-ALA administration were subsequently subjected to ex vivo fluorescence quantification and HHL-PVP incubation.

### 3.2. Fluorescence Intensity Using the Non-Hypericin-Specific Filter

**Figure 2:**
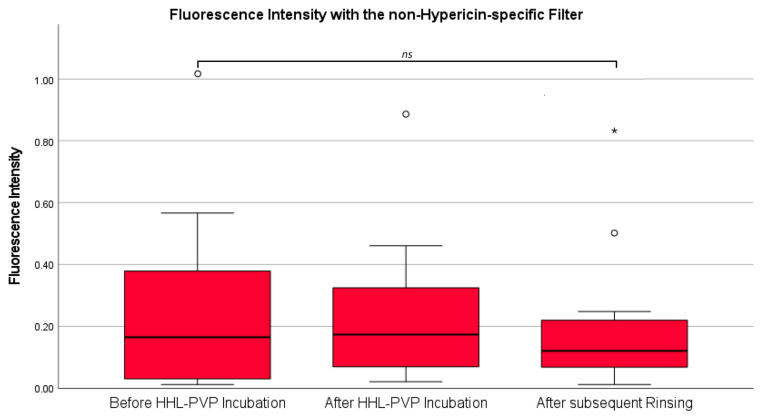
Fluorescence intensity with the non-hypericin-specific filter. Fluorescence intensity across all three time points as observed with the non-hypericin-specific filter is shown in a boxplot diagram. No significant difference across all three time points was found in the ANOVA analysis. ns: not significant. °:outliers. *: far outliers.

**Figure 3:**
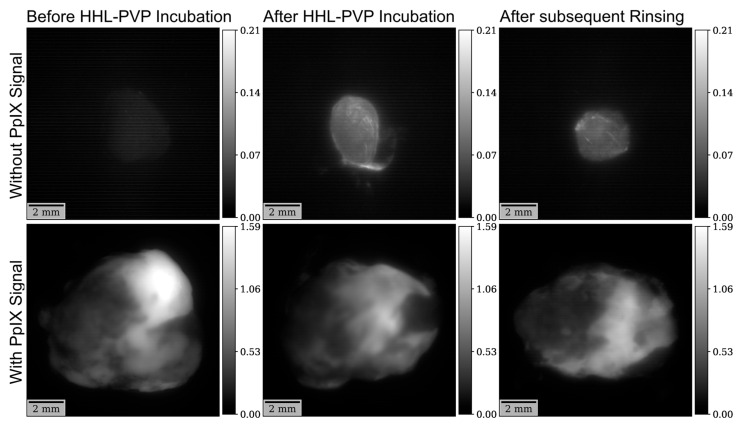
Examples of fluorescence intensity images with the non-hypericin-specific filter. (**Top row**) A clear increase in fluorescence intensity is seen after HHL-PVP incubation as well as after subsequent rinsing compared to the image obtained before incubation in a specimen without 5-ALA-induced PpIX signal. (**Bottom row**) In contrast, due to significant overlying PpIX fluorescence signal, no clear increase in fluorescence intensity could be seen after HHL-PVP incubation in a specimen showing strong 5-ALA-induced fluorescence.

In a first analysis, fluorescence intensity was investigated with a non-hypericin-specific filter previously established for 5-ALA-induced fluorescence visualization in all 12 collected tumor samples. According to our analysis, the mean fluorescence intensity using the non-hypericin-specific filter was 0.27 ± 0.30 at the measurement before HHL-PVP incubation, 0.24 ± 0.26 after HHL-PVP incubation, and 0.20 ± 0.24 after subsequent rinsing. There was overall no significant difference (*p =* 0.876) in the mean fluorescence intensity across all three measurements. A visualization of fluorescence intensity analyses with the non-hypericin-specific filter is provided in Figure 2. Examples of fluorescence intensity images in specimens with and without a 5-ALA-induced PpIX fluorescence signal at all three time points are shown in Figure 3.

### 3.3. Fluorescence Lifetime Using the Non-Hypericin-Specific Filter

**Figure 4:**
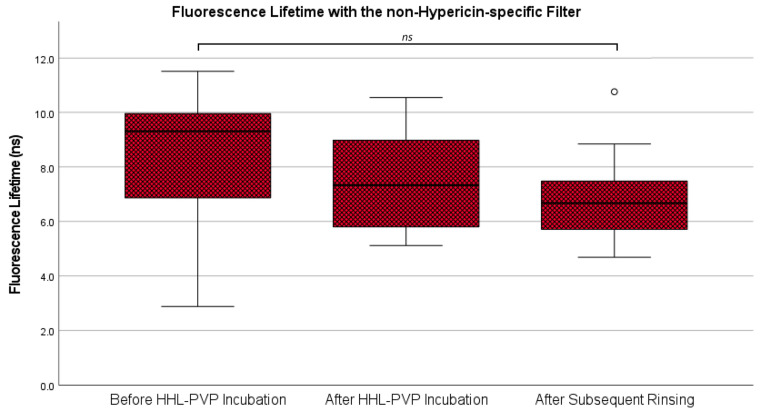
Fluorescence lifetime with the non-hypericin-specific filter. Fluorescence lifetime across all three time points as observed with the non-hypericin-specific filter is shown in a boxplot diagram. No significant difference across all three time points was found in the ANOVA analysis. ns: not significant. °: outliers.

**Figure 5:**
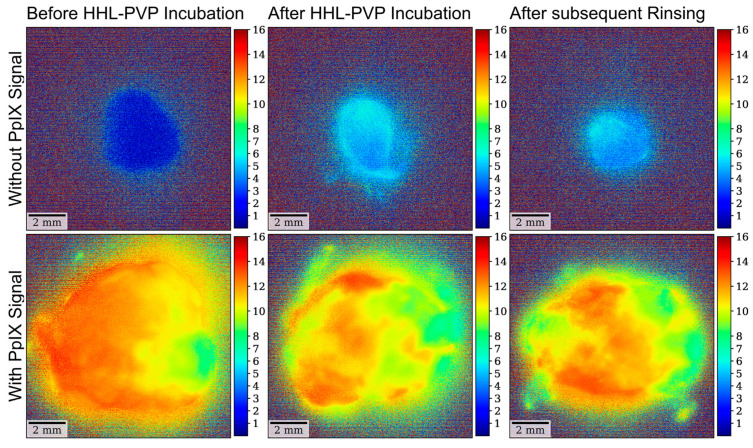
Examples of fluorescence lifetime images with the non-hypericin-specific filter. While a clear increase in fluorescence lifetime can be seen after HHL-PPVP incubation in a specimen without PpIX signal (**top row**), no clear difference is visible in the presence of overlaying PpIX signal (**bottom row**).

In addition to the analysis of fluorescence intensity, the fluorescence lifetime was investigated in all 12 specimens. According to our data, the mean fluorescence lifetime with the non-hypericin-specific filter was 8.31 ± 2.72 ns at the measurement before HHL-PVP incubation, 7.52 ± 1.79 ns after HHL-PVP incubation, and 6.88 ± 1.69 ns after subsequent rinsing. While there was a slow decrease in the mean fluorescence lifetime across the time points of measurement, no statistically significant differences were detected (*p* = 0.270). Figure 4 shows a graphic representation of the fluorescence lifetime analyses with the non-hypericin-specific filter. Examples of fluorescence lifetime images in specimens with and without 5-ALA-induced PpIX fluorescence signals at all three time points are shown in Figure 5.

### 3.4. Fluorescence Intensity Using the Hypericin-Specific Filter

**Figure 6:**
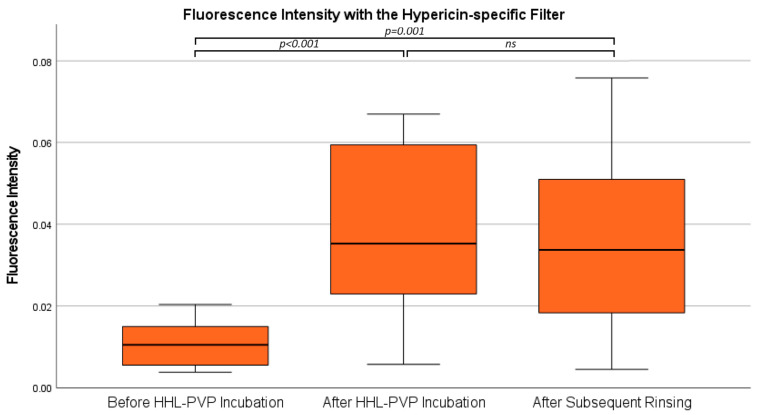
Fluorescence intensity with the hypericin-specific filter. Fluorescence intensity across all three time points as observed with the hypericin-specific filter is shown in a boxplot diagram. Significant differences were present in the ANOVA comparing all three time points, while subsequent paired *t*-tests only demonstrated a significant change after HHL-PVP incubation but not after subsequent rinsing. ns: not significant.

**Figure 7:**
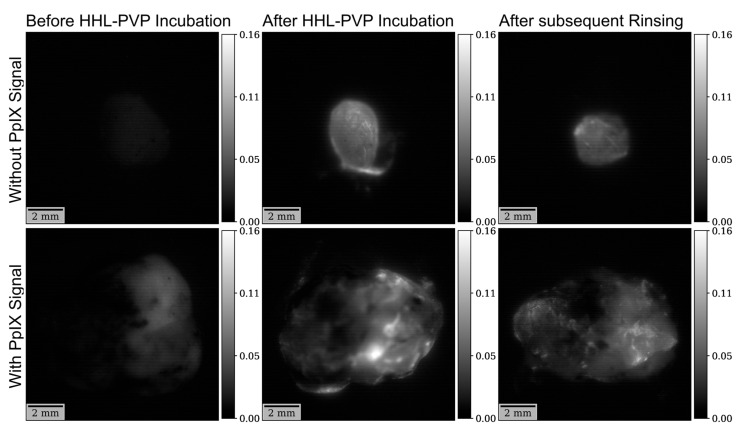
Examples of fluorescence intensity images with the hypericin-specific filter. (**Top row**) A clear increase in fluorescence intensity is seen after HHL-PVP incubation as well as after subsequent rinsing compared to the image obtained before incubation in a specimen without 5-ALA-induced PpIX. (**Bottom row**) With the hypericin-specific filter, a marked increase in fluorescence signal after HHL-PVP incubation can also be seen in a specimen showing strong 5-ALA due to mostly blocked PpIX fluorescence signal.

Moreover, the same analyses were carried out using the narrower bandpass filter for specific hypericin visualization, blocking the PpIX fluorescence peak at 620 nm in all 12 specimens. According to our analysis, the mean fluorescence intensity in the analyses with the hypericin-specific filter was 0.01 ± 0.01 at the measurement before HHL-PVP incubation, 0.04 ± 0.02 after HHL-PVP incubation, and 0.04 ± 0.02 after subsequent rinsing. The overall differences between the three investigated measurements were statistically significant (*p* = 0.001). The subsequent post hoc test to analyze differences between distinct measurements demonstrated a significantly lower fluorescence intensity before HHL-PVP incubation as compared to both subsequent measurements, including after HHL-PVP incubation (*p =* 0.001) and after subsequent rinsing (*p* = 0.003). In contrast, fluorescence intensity did not significantly differ (*p* = 0.876) between the measurements after HHL-PVP incubation and after subsequent rinsing. The results of the fluorescence lifetime analyses with the hypericin-specific filter are shown in Figure 6. Examples of fluorescence intensity images in specimens with and without a 5-ALA-induced PpIX fluorescence signal at all three time points are shown in Figure 7.

### 3.5. Fluorescence Lifetime Using the Hypericin-Specific Filter

**Figure 8:**
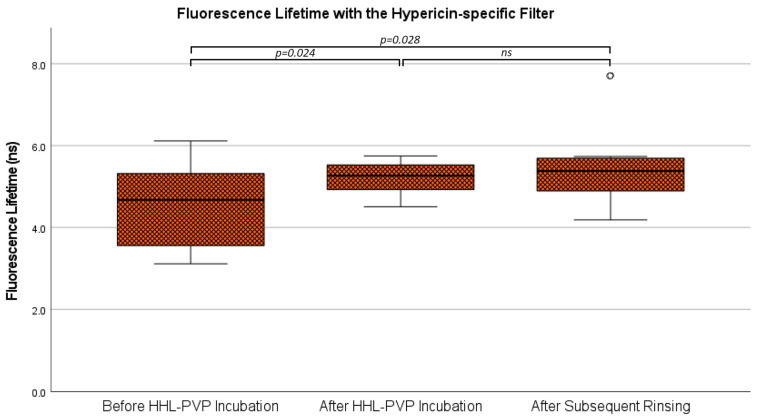
Fluorescence lifetime with the hypericin-specific filter. Fluorescence lifetime across all three time points as observed with the hypericin-specific filter is shown in a boxplot diagram. Significant differences were present in the ANOVA comparing all three time points, while subsequent paired t-tests only demonstrated a significant change after HHL-PVP incubation but not after subsequent rinsing. ns: not significant. °: outliers.

**Figure 9:**
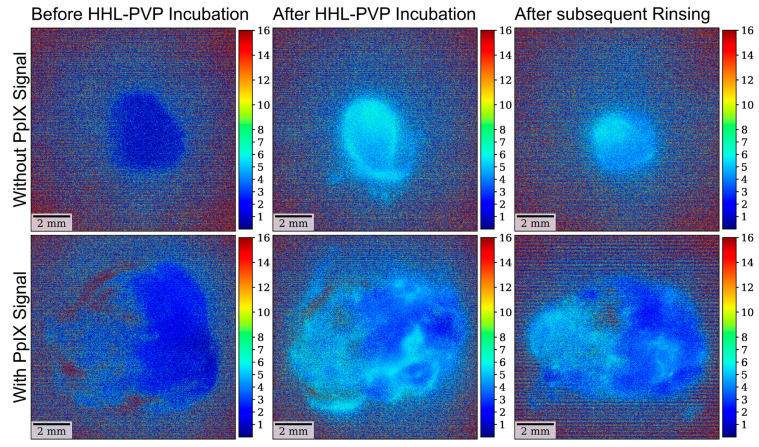
Examples of fluorescence lifetime images with the hypericin-specific filter. A marked increase in fluorescence lifetime can be seen after HHL-PVP incubation in specimens without (**top row**) as well as with a (**bottom row**) significant 5-ALA-induced PpIX fluorescence signal.

Further, fluorescence lifetime was investigated in all 12 specimens with the hypericin specific filter. The mean fluorescence lifetime in the analyses with the non-hypericin-specific filter was 4.51 ± 1.00 ns at the measurement before HHL-PVP incubation, 5.21 ± 0.41 ns after HHL-PVP incubation, and 5.40 ± 0.87 ns after subsequent rinsing. The overall differences between the three investigated measurements were statistically significant (*p =* 0.024). The post hoc test demonstrated significantly lower fluorescence lifetime before HHL-PVP incubation as compared to both subsequent measurements including after HHL-PVP incubation (*p =* 0.039) and after subsequent rinsing (*p =* 0.028). In contrast, no significant difference (*p* = 0.495) was detected between the measurements after HHL-PVP incubation and after subsequent rinsing. A visualization of fluorescence lifetime analyses with the hypericin-specific filter is provided in Figure 8. Examples of fluorescence lifetime images in specimens with and without a 5-ALA-induced PpIX fluorescence signal at all three time points are shown in Figure 9.

### 3.6. Discrimination Between Analyses Before and After Incubation According to Regression Model

**Figure 10:**
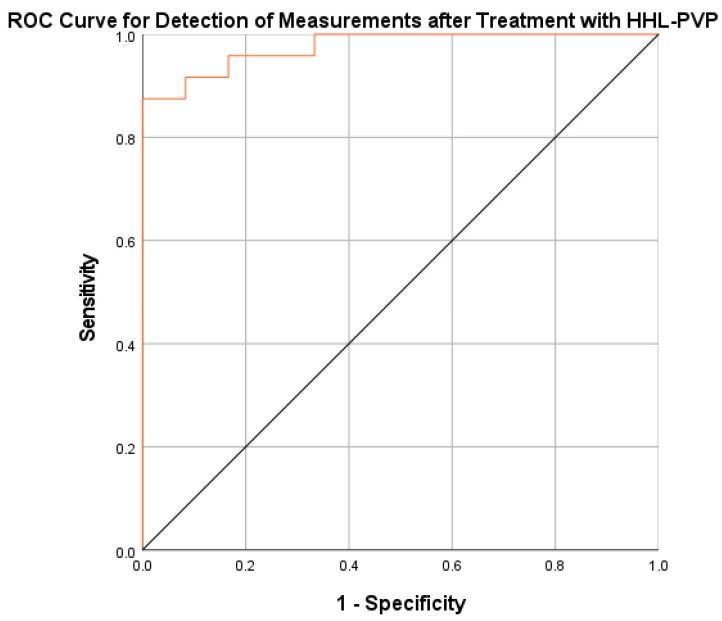
ROC Curve for the detection of measurements after treatment with HHL-PVP.

Finally, we performed a regression model to discriminate between analyses performed without an HHL-PVP signal (before HHL-PVP incubation) or with an HHL-PVP signal (after HHL-PVP incubation and after subsequent rinsing) according to hypericin selective fluorescence intensity and lifetime. According to this model, factors in the equation included mean fluorescence intensity, with a regression coefficient of 550.059, and fluorescence lifetime, with a regression coefficient of 4.595, as well as a constant of −29.159. In the subsequent ROC analysis, this model demonstrated an area under the curve of 97.6% (95% CI: 93.6–100%), corresponding to a sensitivity of up to 87.5% with concurrent specificity of 100%. The ROC curve for the prediction of a measurement with an HHL-PVP signal is shown in Figure 10.

## 4. Discussion

In this study, we analyzed the capability of a previously developed CMOS camera-based system for real-time wide-field fluorescence intensity and lifetime imaging of intraoperatively obtained brain tissue specimens to selectively visualize HHL-PVP in glioblastoma tissue after ex vivo incubation. The development of such techniques for not only reliable but also selective fluorescence detection will be crucial for the future investigation of novel fluorescent dyes in neurosurgery beyond the most widely used fluorescent agent, 5-ALA. Consequently, analyses were performed using a previously established, non-hypericin specific filter (~590–740 nm) designed for visualization of 5-ALA, which also transmits a substantial amount of hypericin fluorescence. Additionally, a newly established hypericin-specific filter (~575–615 nm) was used to selectively visualize HHL-PVP without significant interference from the PpIX signal [34].

### 4.1. Fluorescence Quantification with the Non-Hypericin Specific Filter

In the analyses performed with the previously established bandpass filter transmitting both the fluorescence spectra of 5-ALA-derived PpIX and hypericin, no significant changes in fluorescence intensity or lifetime were observed after HHL-PVP incubation. This is most likely attributable to the significant 5-ALA-induced fluorescence present in almost all specimens. In contrast, in the single specimen obtained from a patient that did not receive 5-ALA prior to surgery, a clear visual difference in the images showing fluorescence lifetime and intensity was present (see also Figure 3 and Figure 5). It is of note that while no significant difference was present, the mean as well as the maximum fluorescence intensity seemed to decrease slightly and consistently with time and even following HHL-PVP incubation. This may be attributable to the bleaching effect of PpIX fluorescence captured with this filter, which outweighed the differences resulting from HHL-PVP accumulation.

### 4.2. Fluorescence Quantification with the Hypericin Specific Filter

In contrast, we observed clear differences in the measurements taken immediately after HHL-PVP incubation as well as after subsequent rinsing compared to the baseline measurement obtained before HHL-PVP incubation. This included significant increases in both fluorescence intensity as well as fluorescence lifetime. These findings demonstrate that our previously developed CMOS camera-based system for real-time wide-field fluorescence imaging upgraded with a narrow bandpass filter at approximately 575–615 nm was capable of reliably and selectively visualizing HHL-PVP fluorescence, successfully blocking 5-ALA-induced fluorescence signals. Further, no difference in either fluorescence intensity or lifetime was found between the measurements obtained after HHL-PVP incubation and subsequent rinsing. On the one hand, this observation is not in accordance with the minimal photobleaching effect of HHL-PVP demonstrated in a previous study [26]. On the other hand, this finding suggests that even after ex vivo incubation, active accumulation within tumor tissue occurs. The observed fluorescence signal thus seems to correspond to actual intratumoral HHL-PVP rather than merely to the HHL-PVP solution on the specimen’s surface. Lastly, according to the regression model that considers fluorescence lifetime and intensity, a high accuracy for discrimination between measurements obtained before and after HHL-PVP incubation—with 87.5% sensitivity and 100% specificity—was possible. It must be pointed out that this analysis was based on the same small sample size of 12 specimens used for the model generation. The results may thus overestimate accuracy, and certainly still need to be confirmed in future independent larger study cohorts. It is nevertheless noteworthy that these values are very similar to the described diagnostic accuracy of hypericin fluorescence for histological glioblastoma tissue previously reported, with a 91–94% sensitivity and 90–100% specificity after the intravenous application of non-PVP-hypericin [22]. It is thus plausible that the minor diagnostic inaccuracy, at least in part, represents biological variance in fluorescence behavior rather than methodological shortcomings in fluorescence quantification.

Interestingly, differences between the measurements obtained before and after HHL-PVP incubation were more pronounced with regard to fluorescence intensity than to fluorescence lifetime. This was somewhat unexpected since the same system previously demonstrated a higher sensitivity towards 5-ALA-induced fluorescence lifetime compared to fluorescence intensity [10]. One likely contributing factor to this observation is the fact that hypericin lifetime has a shorter intrinsic fluorescence lifetime—not exceeding 7 ns compared to native PpIX at 16–17 ns—and thus the difference to background tissue autofluorescence at approximately 2 ms is less pronounced [10,35,36]. Likewise, the average autofluorescence lifetime observed in the wavelength spectrum analyzed with the hypericin-specific filter is longer than in the broader spectrum visualized with the non-hypericin-specific filter, and thus fluorescence lifetime changes compared to the background signal are less pronounced than for 5-ALA-specific analyses [37]. Another potential reason is the comparatively narrow frequency spectrum of the bandpass filter specifically used for selective hypericin visualization. While the selective transmission of the hypericin emission peak improves sensitivity for fluorescence intensity changes by blocking out other interfering fluorescence signals, the amount of fluorescence per pixel that can be factored into lifetime measurements decreases in the absence of hypericin signal. Therefore, especially areas of markedly low fluorescence intensity seem to contain relevant background noise. While this issue was methodologically addressed via a threshold-based denoising approach, the comparatively low inherent fluorescence lifetime of hypericin and the highly optimized filter seem to have resulted in the differing importance of both fluorescence characteristics as compared to PpIX fluorescence. It is of note, however, that this study exclusively included glioblastoma specimens—the amount of autofluorescence transmitted by the hypericin-specific filter could be higher in other brain tumor entities. Since this could result in a decrease in the sensitivity of fluorescence intensity-based analyses compared to lifetime-based analyses in distinct tumor entities, we consider it worthwhile to include both measurements in future analyses. In addition, improvements in hypericin fluorescence lifetime imaging could nevertheless be achieved by optimizing the efficiency of the imaging system and more sensitive dual-tap sensor architectures in future developments.

### 4.3. Clinical Relevance

Considering the favorable diagnostic properties of non-orally bioavailable hypericin formulation that required intravenous application, this novel fluorescent dye constitutes a further highly promising agent for improved brain tumor visualization beyond the widely used 5-ALA [22]. In particular, a 2015 study published by Ritz et al. reported a sensitivity of 91–94% and specificity of 90–100% for glioblastoma tissue detection, which corresponds to an even better diagnostic accuracy than that typically reported for 5-ALA (sensitivity: 82.6%; specificity: 88.9%) as the current gold standard for fluorescence-guided brain tumor surgeries [22,38]. Nevertheless, it is of note that this study analyzed 110 specimens obtained from only five patients, and thus a reliable comparison of the actual diagnostic accuracy of both agents does not seem feasible at this point [22]. However, rather than further improving fluorescence guidance in glioblastoma, the primary relevance of HHL-PVP may well lay in further applications. On the one hand, we hope that this novel photodiagnostic agent might also be able to reliably visualize tumor entities that cannot be sufficiently detected with conventional 5-ALA guidance, such as most low-grade gliomas and a subgroup of brain metastases [39,40]. On the other hand, hypericin has also shown highly promising properties for PDT, with effective inactivation of malignant cells, even with comparatively low light intensities and exposure times that can be achieved at comparatively long excitation wavelength spectra, resulting in improved tissue penetration depth [41,42]. These properties suggest a promising perspective for HHL-PVP, with a dual role in both PDD and PDT, compared to agents such as 5-ALA, which require short wavelength excitation light [18]. While 5-ALA is currently the far better established agent for intraoperative tumor visualization, HHL-PVP could potentially facilitate improvements in PDD of tumors not readily visible with 5-ALA-induced fluorescence, as well as PDT, due to its significantly better tissue penetration [25,26]. Especially in the context of PDT, where patients may simultaneously receive systematic anti-cancer treatments, it needs to be pointed out, however, that hypericin has been shown to inhibit anti-apoptotic proteins and that recent in vitro data has demonstrated interactions between hypericin and the anti-cancer drug doxorubicin [43,44]. Any applications in the postoperative setting will therefore require studies on the compatibility of this method with the chemotherapeutic drugs routinely used in brain tumor patients.

Despite all these highly promising results for hypericin application in photodynamic diagnosis as well as therapy, no further studies on clinical hypericin application for brain tumor visualization have been published thus far. However, its poor oral bioavailability—which previously constituted a major drawback, especially compared to 5-ALA—has meanwhile been overcome by non-covalently binding hypericin to PVP and thus making it water-soluble [26]. Further studies of HHL-PVP are therefore crucial for the future investigation of the clinical applications of this promising fluorescent dye, and reliable visualization techniques will therefore be needed to verify that that this novel formulation delivers the same favorable diagnostic accuracy in an in vivo setting—which includes the influence of factors such as blood flow, tissue oxygenation, and metabolic activity—as that previously shown for non-orally bioavailable hypericin. Given the prominent role of 5-ALA as fluorescent dye routinely used in the surgical treatment of many brain tumor patients, selective HHL-PVP visualization, even in the presence of 5-ALA-induced PpIX fluorescence, is of particular interest. The results of this study demonstrated that the addition of a hypericin-specific bandpass filter transmitting a wave spectrum of ~575–615 allows the reliable and selective visualization of HHL-PVP in a typical glioblastoma patient cohort with the presence of significant 5-ALA-induced fluorescence in most specimens. Once the required safety and efficacy data from in vivo animal models are available to perform clinical studies, the results of this study can therefore contribute to the investigation of HHL-PVP as a photodiagnostic agent without withholding 5-ALA guidance as a well-established and routinely used intraoperative brain tumor visualization technique.

### 4.4. Limitations

A number of limitations should be considered in the interpretation of the results presented within this study: (1) This study was designed to establish the technical feasibility of selective HHL-PVP visualization and was carried out in intraoperatively obtained glioblastoma specimens that were subsequently subjected to ex vivo incubation in HHL-PVP solution. Therefore, no conclusion can be drawn in regard to the diagnostic accuracy of fluorescence signal for the presence of tumor tissue after in vivo HHL-PVP administration, and especially for tumor entities other than glioblastoma. Future in vivo studies are thus needed to establish the clinical reliability of HHL-PVP for the intraoperative visualization of glioblastoma and other brain tumor entities in various clinical settings. (2) This investigation was performed in a small cohort of 12 glioblastoma specimens, primarily aimed at demonstrating the differences in fluorescence lifetime and intensity with the hypericin-specific filter before and after HHL-PVP incubation with adequate power. While differences in regard to fluorescence intensity and lifetime were demonstrable despite the small sample size, the diagnostic accuracy according to the presented ROC analysis in particular needs to be confirmed in future studies with larger cohorts. (3) While the capacity for HHL-PVP visualization was clearly established, it needs to be kept in mind that other hypericin formulations may show slightly varying fluorescence emission spectra and that it thus may not be possible to generalize our findings to every hypericin-based compound. Considering the comparatively narrow transmission bandwidth of the hypericin-specific bandpass filter used in this study, the suitability of the described methodological approach for other hypericin formulations should thus be ascertained prior to adoption.

## 5. Conclusions

In this study, we demonstrated the capability of a CMOS camera-based system upgraded with a hypericin-specific filter to selectively visualize HHL-PVP fluorescence in glioblastoma tissue. The significant differences observed in fluorescence intensity and lifetime after HHL-PVP incubation support the effectiveness of this approach even in the presence of 5-ALA-induced fluorescence, whereas no clear differences were detected with the previously developed system that non-selectively visualizes hypericin as well as PpIX fluorescence. These findings establish a reliable technique for hypericin visualization that will allow for the selective visualization of fluorescence signals, even in patients treated with 5-ALA, to further investigate this promising fluorescent agent. The application of HHL-PVP in addition to 5-ALA will thus combine the advantages of both fluorescence dyes to optimize intraoperative fluorescence detection in the future. Ultimately, this may contribute to a broadened application of fluorescence-guided brain tumor surgery to entities that cannot currently be reliably detected, such as low-grade gliomas.

## Data Availability

The data presented in this study are available on reasonable request from the corresponding author due to privacy considerations in this comparatively small patient cohort.
